# Mutation interactions of BRAF and TP53 define novel prognostic stratification and therapeutic implications in papillary thyroid carcinoma

**DOI:** 10.3389/fendo.2025.1584618

**Published:** 2025-09-29

**Authors:** Li Liu, Fang Wei

**Affiliations:** ^1^ The Operation Room, Shengjing Hospital of China Medical University, Shenyang, China; ^2^ Department of the Seventh General Surgery, The Fourth Affiliated Hospital of China Medical University, Shenyang, China

**Keywords:** papillary thyroid cancer, BRAF, TERT, TP53, TCGA, prognosis

## Abstract

**Background:**

Papillary thyroid carcinoma (PTC) requires improved risk stratification through molecular profiling, yet how mutation interactions shape clinical outcomes remains poorly defined.

**Methods:**

This single-center retrospective study analyzed 72 PTC cases using next-generation sequencing to characterize mutation patterns and pathway evolution, with validation against The Cancer Genome Atlas datasets.

**Results:**

We identified three key molecular features: *BRAF* mutations (47.2%) predicted recurrence risk (p < 0.001), *TP53* mutations (15.3%) were more prevalent in advanced thyroid cancers, and mutual exclusivity between *BRAF* and *RET*/*NRAS* mutations (p < 0.01), defining distinct oncogenic pathways. Paradoxically, *BRAF* mutations correlated with survival improvement (hazard ratio = 0.397), challenging conventional prognostic models. Pathway analysis revealed a potential shift from MAPK dominance in PTC to PI3K/NOTCH activation in advanced thyroid cancers, suggesting targetable vulnerabilities for mTOR inhibitors.

**Conclusion:**

By integrating *BRAF*/*TP53* status with conventional staging, we establish a mutation-guided framework that may refine risk prediction and inform treatment strategies, bridging molecular heterogeneity with clinical decision-making. This work provides insights for personalizing thyroid cancer management.

## 1 Introduction

Thyroid cancer is the most common endocrine malignancy, accounting for 3%–4% of all cancer diagnoses globally (1). Over the past few decades, its incidence has steadily increased, making it the fastest-growing solid tumor worldwide (2). In China, cases have surged by 289.6% from 1990 to 2019, particularly among individuals aged 15–49 (3). Papillary thyroid carcinoma (PTC), representing approximately 84% of thyroid malignancies, typically demonstrates favorable outcomes. However, significant challenges persist in predicting recurrence and personalizing treatment strategies. Current prognostic models, which predominantly rely on clinical parameters such as age, tumor size, and histological characteristics, remain limited by their inability to adequately account for the molecular complexity of thyroid cancer pathogenesis (4). The genes *BRAF, TP53*, and *TERT* play critical roles in the molecular pathogenesis of thyroid cancer. Mutations in the *BRAF* gene, particularly the V600E variant, occur in up to 60% of PTC cases and are associated with aggressive tumor features and increased recurrence risk, contributing to tumor initiation and progression through aberrant activation of the MAPK signaling cascade (5). In contrast, *TP53* mutations, which disrupt the function of the p53 tumor suppressor protein and diminish cell cycle and DNA repair mechanisms, are more commonly found in poorly differentiated thyroid carcinoma (PDTC) or anaplastic thyroid carcinomas (ATC) and are strongly associated with adverse clinical outcomes (6). *TERT* promoter mutations upregulate telomerase activity, enabling cancer cells to bypass senescence and promoting unlimited cell division (7). Notably, *TERT* promoter mutations often co-occur with *BRAF* mutations, further exacerbate this risk and are linked to lower overall survival rates across various histological subtypes of thyroid cancer (8). Other mutations, such as those in *RAS* and *RET*, also play crucial roles in the pathogenesis of various thyroid cancer subtypes, affecting their clinical behavior and therapeutic responses (9). Therefore, integrating genetic data with clinical characteristics is needed to enhance patient outcomes by improving histology-based risk stratification and informing treatment decisions (10).

Despite advancements in genomic profiling, several gaps remain in understanding how different genetic mutations interact and collectively impact disease progression. Additionally, reliable biomarkers for accurately predicting patient outcomes are still lacking (11). These challenges hinder the translation of genetic findings into clinical practice and the development of tailored treatments based on individual genetic profiles.

To address these knowledge gaps, we conducted an integrated analysis of genetic mutations and clinical outcomes in patients with PTC. The primary objective was to explore novel associations between specific mutations and clinical outcomes, including recurrence and survival. By examining these relationships, our findings aim to inform the development of more precise prognostic tools and guide future research on personalized treatment strategies in PTC.

## 2 Materials and methods

### 2.1 Study subjects and eligibility criteria

Papillary thyroid cancer patients who underwent surgical treatment at the Department of the Seventh General Surgery, The Fourth Affiliated Hospital of China Medical University, between January 2013 and October 2019, were selected for this study. Ethical approval was granted by the institutional Ethics Committee of the Fourth Affiliated Hospital of China Medical University, ensuring adherence to the principles of the Declaration of Helsinki. Informed consent was obtained from all participants prior to inclusion in the study.

After rigorous screening, 72 patients with identified gene mutations who had undergone surgical treatment were included for in-depth analysis. Postoperatively, patients received routine thyroid-stimulating hormone (TSH) suppression therapy to control disease progression and reduce the risk of recurrence. Additionally, in accordance with the risk stratification criteria outlined in the American Thyroid Association guidelines (12), some patients received radioactive iodine (^131^I) therapy. This comprehensive treatment approach was designed to provide optimal therapeutic outcomes and ensure the accuracy of the data used in the study.

Patients were included based on the following strict criteria: newly diagnosed with thyroid cancer and untreated with radioactive iodine therapy (^131^I), radiofrequency ablation, or any other adjunctive therapies; underwent total or subtotal thyroidectomy with postoperative pathological confirmation of thyroid cancer; complete data accessible through the electronic medical record system; and aged between 18 and 84 years. Exclusion criteria were: incomplete preoperative or postoperative clinical records; presence of other malignancies; severe chronic diseases such as hypertension, heart disease, diabetes, or other significant organic diseases; non-adherence to standardized follow-up treatment protocols after discharge; unclear or disputed pathological diagnosis of thyroid cancer; absence of next-generation sequencing (NGS) for gene mutation detection; cases confirmed as metastatic, benign, indeterminate, borderline, or *in situ* thyroid cancer; endocrine-related disorders; and psychiatric illnesses.

### 2.2 Data collection

General patient information, including age and gender, was collected. Clinical presentation details, such as symptoms at diagnosis and their duration, were documented, along with thyroid function test results [(TSH; parathyroid hormone (PTH); free triiodothyronine (FT3); free thyroxine (FT4); thyroid peroxidase antibody (TPOAb); thyroglobulin antibody (TGAb)]. Details of surgical methods, the extent of lymph node dissection, and any intraoperative or postoperative complications were recorded. Pathological data, including maximum tumor diameter, presence of central or lateral lymph node metastasis, bilateral involvement, and multifocal lesions, were collected. Pathological staging was conducted according to the 8th edition of the American Joint Committee on Cancer (AJCC) staging system (13).

The follow-up period ranged from 22 to 121 months, with a median follow-up time of 55.5 months. During follow-up, key information such as postoperative ^131^I treatment, regular thyroid function tests, ultrasound examinations, and recurrence time was collected. The last telephone follow-up was conducted on November 1, 2023. The results of gene mutation detection obtained through NGS sequencing were also collected.

### 2.3 Criteria for recurrence

Recurrence was diagnosed based on the detection of new nodules or masses in the thyroid bed or neck region through high-resolution neck ultrasound, computed tomography (CT), or magnetic resonance imaging (MRI), exhibiting typical characteristics of thyroid cancer, such as irregular shape, heterogeneous echogenicity, and indistinct margins. Suspicious nodules or masses identified through imaging were subjected to fine-needle aspiration (FNA) for cytological or pathological examination. A significant increase in serum thyroglobulin (Tg) levels (>10 ng/mL measured by immunoassay with functional sensitivity ≤0.1 ng/mL) served as a biochemical indicator of recurrence. To minimize TgAb interference, all Tg measurements were concurrently tested for TgAb using electrochemiluminescence immunoassay (ECLIA). Cases with TgAb positivity (>40 IU/mL) were excluded from Tg-based recurrence assessment (14). For TgAb-negative or low-TgAb cases (<40 IU/mL), elevated Tg levels were considered clinically significant. Additionally, abnormal elevations in tumor markers (e.g., calcitonin for medullary carcinoma) or discrepant Tg/TgAb trends (rising Tg with stable/declining TgAb) were integrated into recurrence evaluation.

### 2.4 Sample preparation, DNA extraction, and sequencing library preparation

Formalin-fixed paraffin-embedded (FFPE) tissue specimens from all patients were subjected to NGS by Nanjing Geneseeq Technology Inc. Genomic DNA was extracted using the QIAamp DNA FFPE Tissue Kit (Qiagen, Germany) following the manufacturer’s instructions. Sequencing libraries were prepared using the KAPA Hyper Prep Kit (KAPA Biosystems, USA). Custom xGen Lockdown probes (Integrated DNA Technologies, USA) were used for hybrid capture targeting 437 tumor-related genes (GeneseeqPrime™ Pan-cancer gene panels; Geneseeq Technology Inc., China).

Hybridization reactions were performed using Dynabeads M-270 (Life Technologies, USA) and the xGen Lockdown Hybridization and Wash Kit (Integrated DNA Technologies, USA) according to the manufacturer’s protocol. The captured libraries were amplified by polymerase chain reaction (PCR) using Illumina p5 (5′-AAT GAT ACG GCG ACC ACC GA-3′) and p7 primers (5′-CAA GCA GAA GAC GGC ATA CGA GAT-3′) in KAPA HiFi HotStart ReadyMix (KAPA Biosystems, USA), followed by purification with Agencourt AMPure XP beads (Beckman Coulter, USA). Library quantification was performed using quantitative PCR (qPCR) with the KAPA Library Quantification Kit (KAPA Biosystems, USA). Library fragment size was determined using the Bioanalyzer 2100 (Agilent Technologies, USA).

Target-enriched libraries were sequenced on the HiSeq 4000 NGS platform (Illumina, USA) according to the manufacturer’s instructions. The average coverage sequencing depth for FFPE tissue samples was 1000×.

### 2.5 Sequencing data analysis

Quality control of the raw sequencing data was performed using Trimmomatic version 0.39 (15). Sequencing adapters and low-quality bases (Phred score <20) were trimmed from the reads, and reads shorter than 36 bp after trimming were discarded. Reads containing ambiguous bases (N bases) were removed. High-quality cleaned reads were aligned to the reference human genome (hg19/GRCh37) using the Burrows-Wheeler Aligner (BWA) MEM algorithm version 0.7.18 (16) with default parameters.

Post-alignment processing was conducted using the Genome Analysis Toolkit (GATK) version 4.1.0.0 (17). This included marking duplicates using Picard tools (version 2.18.14), realignment around indels, and base quality score recalibration. Data normalization and quality control measures were applied to ensure accurate variant calling, including assessment of sequencing depth and uniformity, mapping quality, and duplication rates.

Somatic mutations, including single nucleotide variants (SNVs) and small insertions and deletions (indels), were identified using MuTect2 (GATK version 4.1.0.0) (18) and VarScan2 version 2.4.2 (18) with default parameters. Variants were filtered based on quality scores, read depth, and strand bias. The variants were annotated using ANNOVAR software version 2018Apr16 (19) to identify known and novel mutations, determine their functional effects, and compare them against various databases such as dbSNP (build 151), 1000 Genomes Project, COSMIC v91, and ClinVar.

Copy number variations (CNVs) were detected using CNVkit version 0.9.6 (20), and structural variants (SVs) were identified using Manta version 1.6.0 (21). CNVkit analysis was performed using a reference built from pooled normal samples, and thresholds for copy number gains and losses were set according to standard guidelines. Variants were further filtered to exclude common polymorphisms (allele frequency >1% in the 1000 Genomes Project or ExAC databases) and those with low allele frequencies (<5%).

Pathway enrichment analyses were conducted using the Kyoto Encyclopedia of Genes and Genomes (KEGG) release 91.0 (22) and Reactome database version 70 (23) to assess the functional impact of the identified mutations. The potential clinical significance of the detected mutations was evaluated based on their presence in cancer-related genes and pathways, as well as their known associations with thyroid cancer prognosis and treatment response (24).

### 2.6 Comprehensive analysis of large-scale thyroid cancer genomics

An integrative analysis of mutations exceeding 10% prevalence in the PTC cohort, including *BRAF*, *TERT, RET, TP53, CDKN2A*, and *NRAS*, was performed using cBioPortal (https://www.cbioportal.org/) (25). The datasets Papillary Thyroid Carcinoma (PTC, TCGA, Cell 2014) and Poorly-Differentiated and Anaplastic Thyroid Cancers (PDTC/ATC, MSK, JCI 2016) (26) were selected. The mutual exclusivity and co-occurrence of these mutations and their correlation with survival time, were assessed according to cBioPortal’s instructions. The curated thyroid cancer pathways invloved the mutations were outlined using PathwayMapper 2.3 (https://www.pathwaymapper.org/) (27). The functional enrichment was analyzed and visualized using ShinyGO 0.81 (https://bioinformatics.sdstate.edu/go/) (28) and STRING 12.0 (https://string-db.org/) (29).

### 2.7 Statistical analysis

Data analysis was performed using SPSS statistical software version 22.0 (IBM Corp., USA). Continuous variables were expressed as mean ± standard deviation (mean ± SD) and compared using independent-samples t-tests. Categorical variables were expressed as frequencies and percentages (%) and compared using Chi-square tests or Fisher’s exact test where appropriate. Univariate and multivariate Cox proportional hazards regression analyses were employed to evaluate the associations between clinical characteristics, genetic mutations, and prognosis. Hazard ratios (HRs) with 95% confidence intervals (CIs) were calculated. Kaplan-Meier survival curves were generated to assess relapse-free survival (RFS), and differences were evaluated using the log-rank test. In all statistical analyses, a p-value <0.05 was considered statistically significant.

Due to the retrospective nature of the study and incomplete clinical documentation, data regarding adjuvant radioactive iodine (RAI) therapy and the completeness of surgical resection were not consistently available and were therefore excluded from the multivariate models. Although all patients underwent curative thyroidectomy, detailed surgical margin status and adjuvant treatment records were not uniformly reported.

## 3 Results

### 3.1 Mutation profiles across the PTC cohort and TCGA datasets

**Figure 1:**
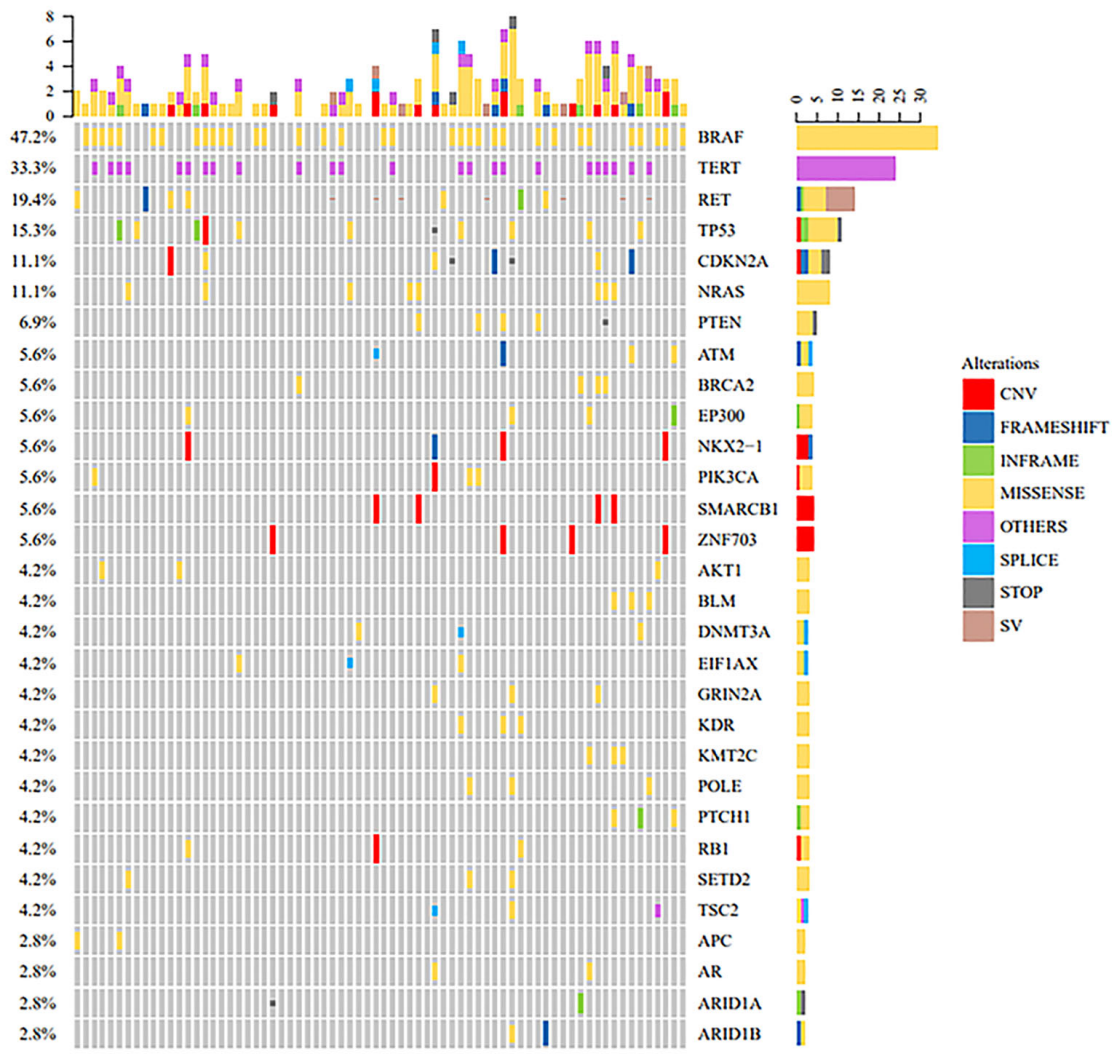
Mutation landscape of 72 papillary thyroid cancer cases. This oncoprint diagram presents the mutation profiles of the papillary thyroid carcinoma (PTC) cohort, highlighting the most recurrently mutated genes. Each column represents an individual case, while rows represent genes. Different colors indicate specific mutation types. The top bar graph illustrates the mutation burden per sample, and the right panel summarizes the mutation prevalence of each gene.

**Figure 2:**
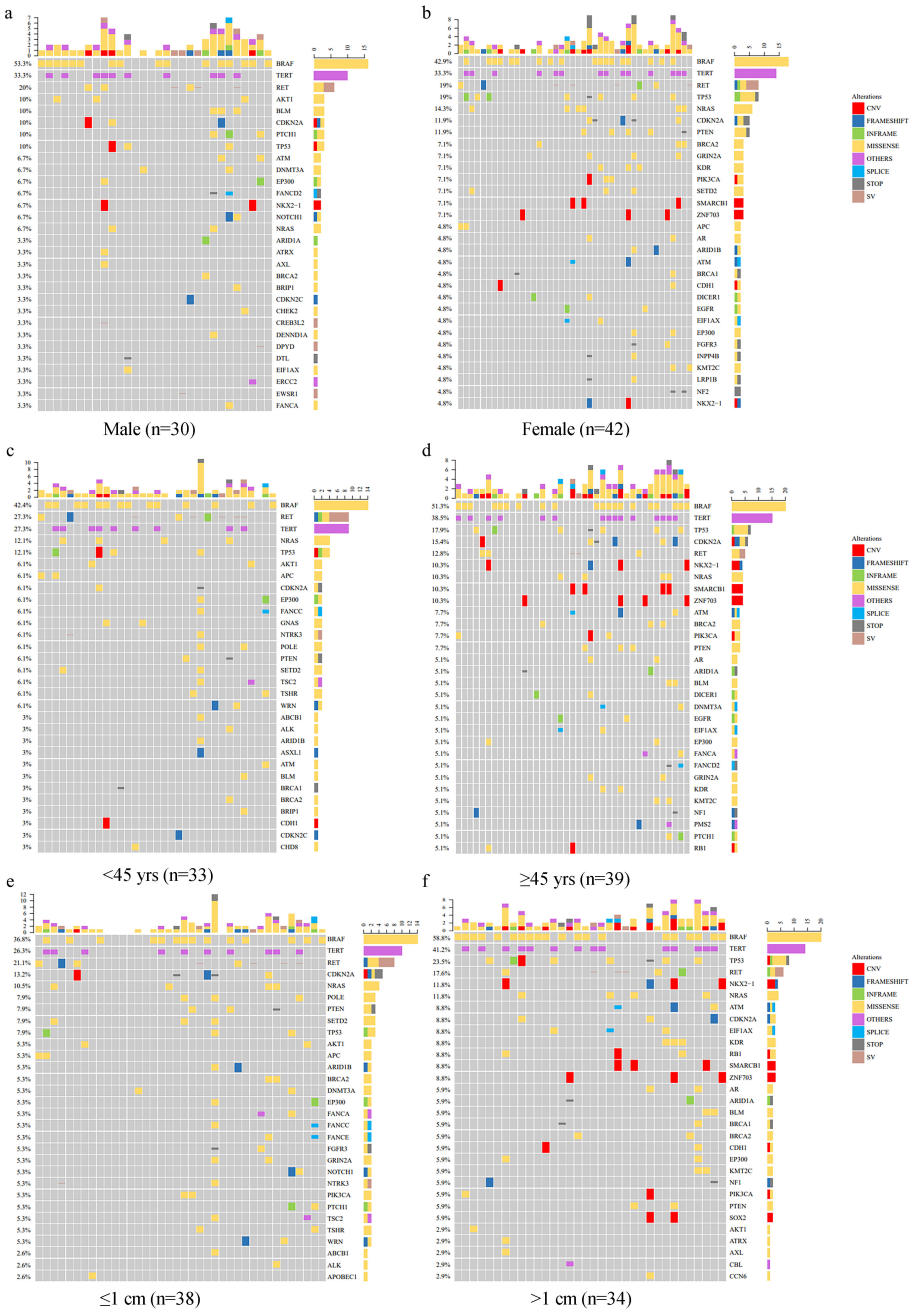
Mutation profiles of PTC clinical subgroups. Oncoprint diagrams showing the mutation profiles of PTC cases stratified by clinical subgroups, including: **(a)** Male; **(b)** Female; **(c)** Age < 45 years; **(d)** Age ≥ 45 years; **(e)** Tumor size ≤ 1 cm; **(f)** Tumor size > 1 cm. Each panel illustrates the mutational landscape per subgroup, with the most frequent mutations displayed in descending order of frequency. Different colors represent distinct mutation types.

Thyroid cancer exhibits substantial molecular heterogeneity, and understanding the genetic landscape of Papillary Thyroid Carcinoma (PTC) is critical for improving diagnostic and therapeutic strategies. To characterize the mutational profiles, we analyzed genetic alterations in 72 PTC cases and compared their mutation distributions across different clinical subgroups and The Cancer Genome Atlas (TCGA) datasets. The clinicopathological characteristics of the cohort are summarized in **Table 1**, showing a balanced distribution of demographic and clinical parameters, with 58.3% of cases being female (42/72), 54.2% aged ≥45 years (39/72), and 37.5% experiencing recurrence (27/72). The mutational landscape of our cohort (**Figure 1**) revealed that *BRAF* was the most frequently mutated gene (47.2%), followed by *TERT* (33.3%), *RET* (19.4%), *TP53* (15.3%), *CDKN2A* (11.1%), and *NRAS* (11.1%). The mutation burden varied among tumors, with some harboring only a single driver mutation, while others displayed multiple co-occurring alterations. Further subgroup analyses revealed differential mutation prevalence across clinical categories (**Figure 2**). *BRAF* mutations occurred in 53.3% of males versus 42.9% of females, 42.4% of patients <45 years versus 51.3% ≥45 years, and 36.8% of tumors ≤1 cm versus 58.8% >1 cm. Similarly, *TERT* mutations showed varying prevalence by age (27.3% <45 years vs. 38.5% ≥45 years) and tumor size (26.3% ≤1 cm vs. 41.2% >1 cm). *RET* mutations were more frequent in younger patients (27.3% vs. 12.8%), while *TP53* mutations increased with age (10% vs. 19%) and larger tumor size (7.9% vs. 23.5%). However, these differences were not statistically significant (**Table 2**).

**Table 1:** Clinicopathological characteristics of 72 papillary thyroid cancer cases.

Clinicalphathological features	Cases (%)
Gender	
male	30 (41.67%)
female	42 (58.33%)
Age	
≥45	39 (54.17%)
<45	33 (45.83%)
Maximum tumor diameter (cm)	
≤ 1	38 (52.78%)
>1	34 (47.22%)
Number of tumor foci	
≥1	33 (45.83%)
1	39 (54.17%)
Lymph node metastasis	
positive	10 (13.89%)
negative	62 (86.11%)
AJCC TNM staging	
I	47 (65.28)
II	19 (26.39%)
III	2 (2.78%)
IV	4 (5.55%)
Recurrence	
negative	45 (62.5%)
positive	27 (37.5%)

**Table 2:** Associations between key driver mutations and recurrence.

Mutations		Gender				Age				Tumor size				Recurrence			
Gene	Status	Male	Female	χ2	P value	<45	≥45	χ2	P value	≤2	>2	χ2	P value	Yes	No	χ2	P value
*BRAF*	MUT	16	18	0.771	0.380	14	20	0.563	0.453	14	20	3.479	0.062	19	15	12.726	<0.001
	WT	14	24			19	19			24	14			6	32		
*TERT*	MUT	10	14	<0.001	>0.9999	19	27	1.007	0.316	10	14	1.783	0.182	12	12	3.707	0.050
	WT	20	28			14	12			28	20			13	35		
*RET*	MUT	6	8	0.010	0.919	9	5	2.384	0.123	8	6	0.133	0.716	3	11	1.355	0.244
	WT	24	34			24	34			30	28			22	36		
*TP53*	MUT	3	8	NA	0.069	4	7	0.469	0.494	3	8	3.389	0.066	8	3	8.270	<0.001
	WT	27	34			29	32			35	26			17	44		
*CDKN2A*	MUT	3	5	0.064	0.800	2	6	1.573	0.210	5	3	0.341	0.559	2	6	0.375	0.540
	WT	27	37			31	33			33	31			23	41		
*NRAS*	MUT	2	6	NA	0.169	4	4	0.063	0.802	4	4	0.028	0.867	3	5	0.031	0.861
	WT	28	36			29	35			34	30			22	42		

MUT, Mutation; WT, wild-type. The differences of *TP53* and *NRAS* in the gender subgroups were examined using Fisher’s exact test. A p-value <0.05 was considered statistically significant. NA, Not applicable.

**Figure 3:**
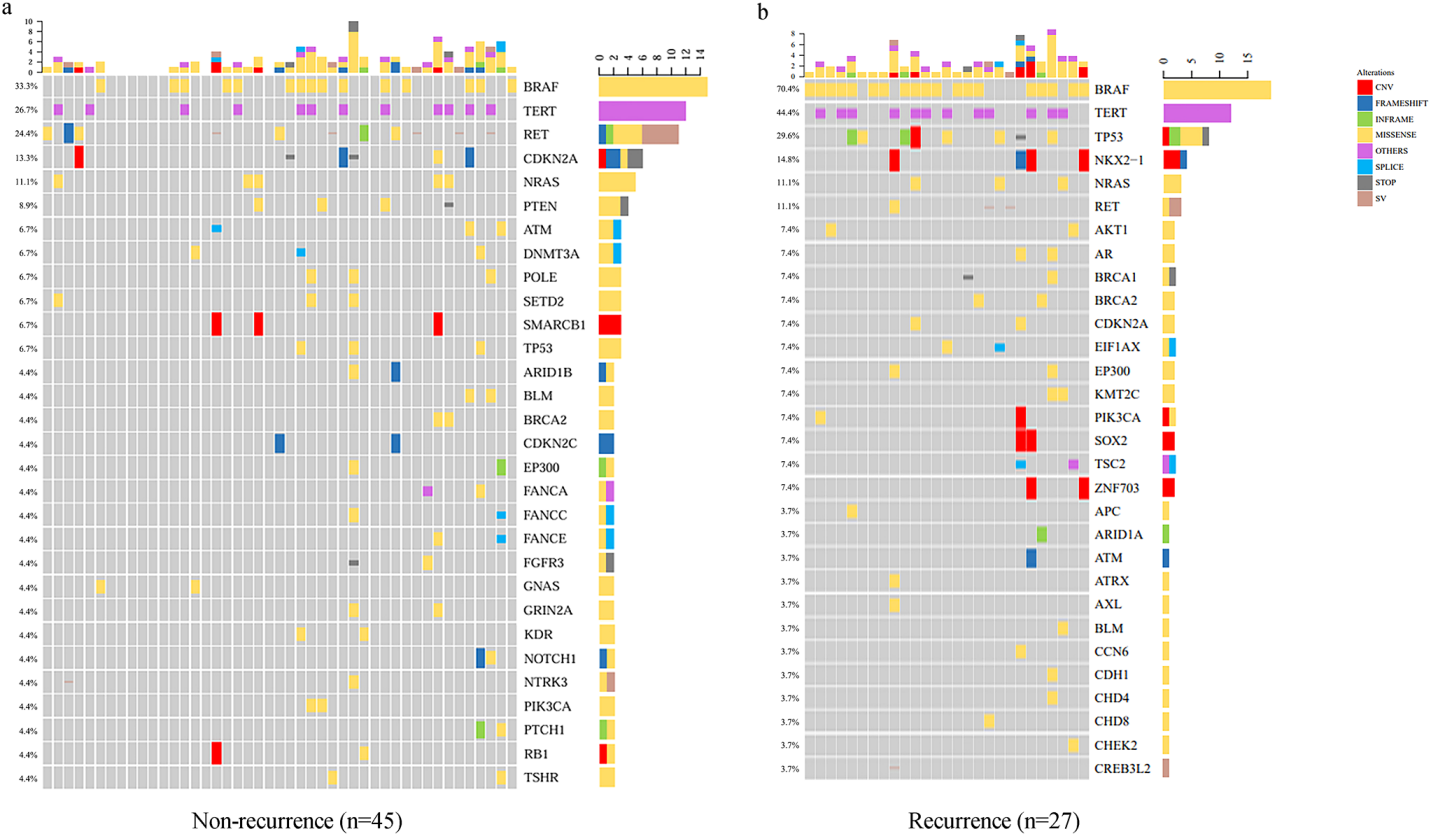
Mutation profiles in recurrent and non-recurrent PTC patients. Oncoprint diagrams comparing the mutational landscape of non-recurrent and recurrent PTC cases: **(a)** Non-recurrent PTC cases; **(b)** Recurrent PTC cases. Key differences in mutation prevalence and distribution are highlighted, with *BRAF*, *TERT*, and *TP53* mutations observed more frequently in recurrent cases.

Notably, recurrent cases demonstrated substantially higher *BRAF* (70.4% vs. 33.3%) and *TERT* (44.4% vs. 26.7%) mutation frequencies compared to non-recurrent cases, but lower *RET* (11.1% vs. 24.4%) and *TP53* (29.6% vs. 6.7%) prevalence (**Figure 3**). Both *BRAF* and *TP53* mutations showed strong correlations with recurrence (p < 0.001), underscoring their prognostic relevance. Although *TERT* mutations did not reach statistical significance (p = 0.050), the observed trend warrants further investigation. No significant associations were observed for *RET*, *CDKN2A*, or *NRAS* mutations.

These findings align with the observed mutation distributions across clinical subgroups (**Figures 1**–**3**), reinforcing the notion that *BRAF* and *TP53* mutations are key contributors to recurrence risk in PTC. The lack of significant associations for other mutations, such as *NRAS* and *CDKN2A*, suggests that their roles in PTC pathogenesis may be context-dependent or influenced by additional molecular alterations. By comparing our cohort with TCGA datasets (**Supplementary Figure 1**), we observed that the prevalence of *BRAF* mutations was relatively consistent between our cohort (47.2%) and TCGA-PTC (50.0%), whereas *TP53* mutations were significantly more frequent in TCGA-PDTC/ATC (28%) than in TCGA-PTC (0.8%) or our cohort (15.3%). This pattern highlights the potential role of *TP53* in tumor dedifferentiation and progression to more aggressive thyroid cancer subtypes.

Collectively, these findings suggest that while *BRAF* mutations are central to PTC initiation, *TP53* and *TERT* mutations may be more relevant in recurrence and disease progression. The distinct mutational landscapes observed in PDTC/ATC further indicate shifts in oncogenic pathways during thyroid cancer evolution, which will be examined in the next section through mutation correlation analysis.

### 3.2 Mutation correlations, protein-protein interactions, and pathway alterations in thyroid cancer

**Figure 4:**
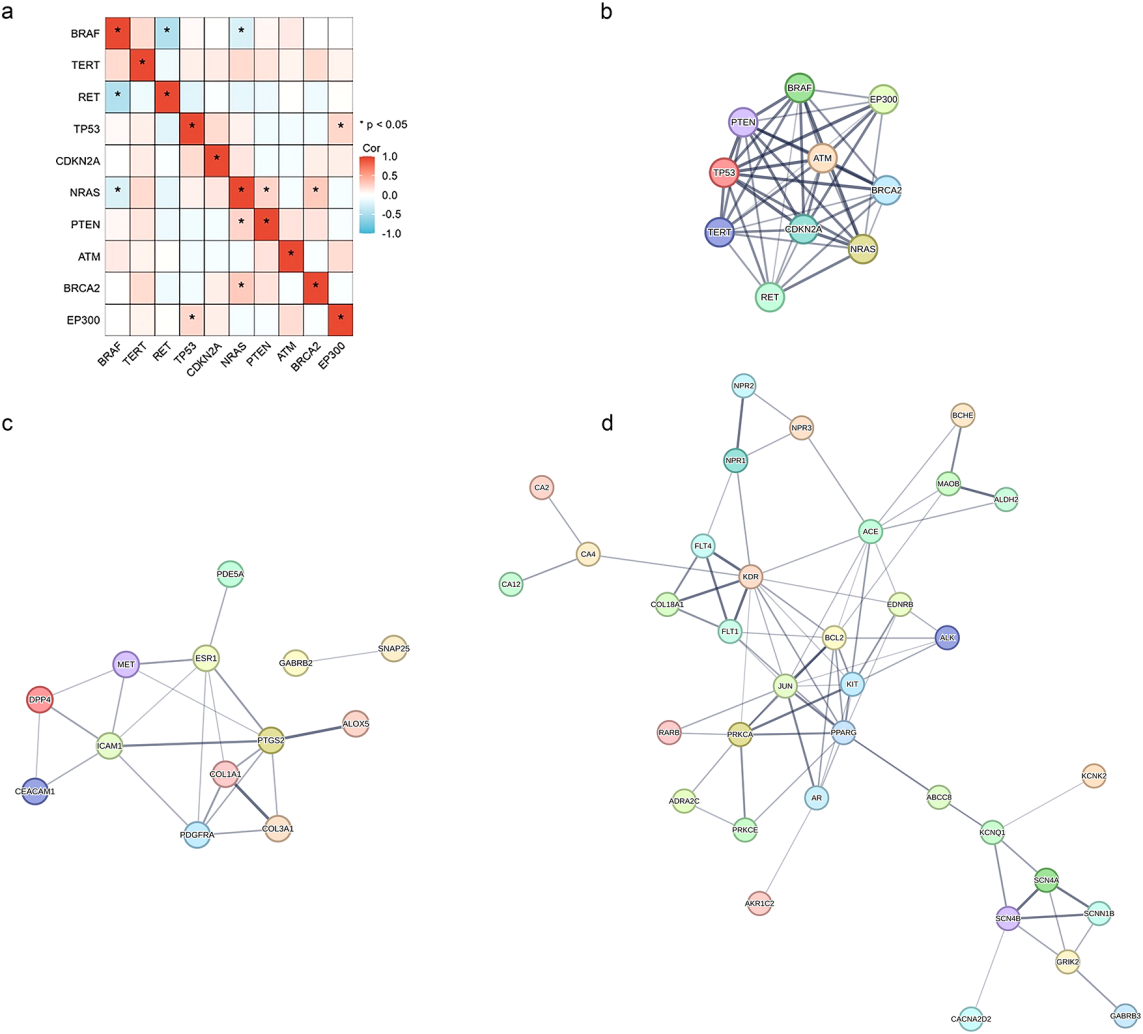
Mutation correlations and protein-protein interaction (PPI) networks in papillary thyroid cancer. This figure presents the mutation correlation matrix and PPI networks of key mutated genes and FDA-approved drug target genes in PTC and TCGA datasets: **(a)** Mutation correlation matrix of the top 10 frequently mutated genes in the 72 PTC cases. Significant co-occurrences are shown in red, while mutually exclusive relationships are in blue (p < 0.05). **(b)** PPI network of the top 10 frequent mutations in PTC, illustrating functional interactions among driver mutations. **(c)** PPI network of the upregulated FDA-approved drug target genes in TCGA datasets, highlighting oncogenic signaling pathways. **(d)** PPI network of the downregulated FDA-approved drug target genes in TCGA datasets, indicating loss of tumor suppressor functions. These findings suggest that various co-mutation patterns and drug target alterations may drive thyroid cancer progression and contribute to distinct therapeutic responses.

Analysis of mutation correlations in 72 papillary thyroid cancer (PTC) cases revealed distinct patterns of co-occurrence and mutual exclusivity. Significant co-mutations were identified between *TP53* and *EP300*, *NRAS* and *PTEN*, as well as *NRAS* and *BRCA2*, suggesting potential cooperative oncogenic mechanisms. In contrast, *BRAF* mutations displayed mutual exclusivity with *RET* and *NRAS* mutations, reinforcing their distinct roles as alternative oncogenic drivers in PTC (**Figure 4a**). The interaction of the proteins were further investigated. A protein-protein interaction network highlighted interactions at the protein level (**Figure 4b**).

To validate these observations, mutation correlations were further examined in the TCGA-PTC and TCGA-PDTC/ATC datasets (**Supplementary Tables 1**, **2**). The exclusivity of *BRAF* with *RET* and *NRAS* was consistently observed in TCGA-PTC, supporting its robustness across different cohorts. However, the co-mutations involving *TP53*, *NRAS*, *PTEN*, and *BRCA2*, which were evident in our dataset, were not observed in either TCGA-PTC or TCGA-PDTC/ATC. This discrepancy suggests that these alterations may be enriched in a subset of PTCs with specific clinicopathological characteristics. Additionally, *BRAF*-*TERT* co-occurrence was observed across all three datasets but did not reach statistical significance.

To gain further insights into the functional impact of these genetic alterations, we explored FDA-approved drug target genes affected by the five most frequent mutations in TCGA-PTC (**Supplementary Table 3**) and TCGA-PDTC/ATC (**Supplementary Table 4**), respectively. The identified drug target genes exhibited distinct expression patterns in each dataset, prompting further investigation into their functional interactions. The PPI network of upregulated drug target genes in both TCGA-PTC and TCGA-PDTC/ATC datasets (**Figure 4c**) revealed their clustering within oncogenic signaling pathways, indicating potential therapeutic vulnerabilities. Conversely, the PPI network of downregulated drug target genes in both datasets (**Figure 4d**) demonstrated their involvement in tumor suppressor pathways, suggesting a loss of tumor-suppressive functions. These findings highlight the relevance of mutation-driven alterations in drug target genes and their potential influence on therapeutic response.

Further comparative pathway analysis of the TCGA-PTC and TCGA-PDTC/ATC datasets underscored distinct differences in the activation of key oncogenic pathways (**Supplementary Figure 2**). PTC tumors generally retained functional *TP53*/*CDKN2A* signaling, which likely contributed to controlled tumor growth and more favorable outcomes. In contrast, PDTC/ATC exhibited frequent *TP53* and *CDKN2A* mutations, promoting genomic instability, aggressive progression, and therapy resistance. Similarly, *BRAF* mutations were predominant in PTC, reinforcing well-differentiated tumor characteristics, whereas PDTC/ATC showed a transition toward *NRAS*-driven oncogenesis, associated with poorer differentiation and altered therapeutic responses.

In terms of cell cycle regulation, PTC tumors displayed minimal disruption of *CDKN2A* and *RB1*, preserving controlled proliferation. However, PDTC/ATC exhibited substantial deregulation of these pathways, leading to unchecked cell cycle progression and reduced sensitivity to checkpoint inhibitors. Moreover, PI3K pathway activation was relatively uncommon in PTC but frequently altered in PDTC/ATC, signifying a shift toward PI3K-driven survival mechanisms and increased resistance to targeted therapies. Lastly, NOTCH signaling remained largely intact in PTC, supporting differentiation programs, while PDTC/ATC demonstrated widespread NOTCH pathway disruption, favoring dedifferentiation, metastasis, and more aggressive disease progression.

In summary, these findings illustrate the molecular divergence between well-differentiated PTC and its more aggressive counterparts, PDTC and ATC. The identification of co-mutation patterns, pathway alterations, and drug target gene disruptions suggests distinct mechanisms driving tumor behavior and therapeutic responses. These insights may aid in refining personalized treatment strategies and identifying novel therapeutic targets for more aggressive thyroid cancer subtypes.

### 3.3 Association between clinicopathological features, mutations, and survival outcomes

**Figure 5:**
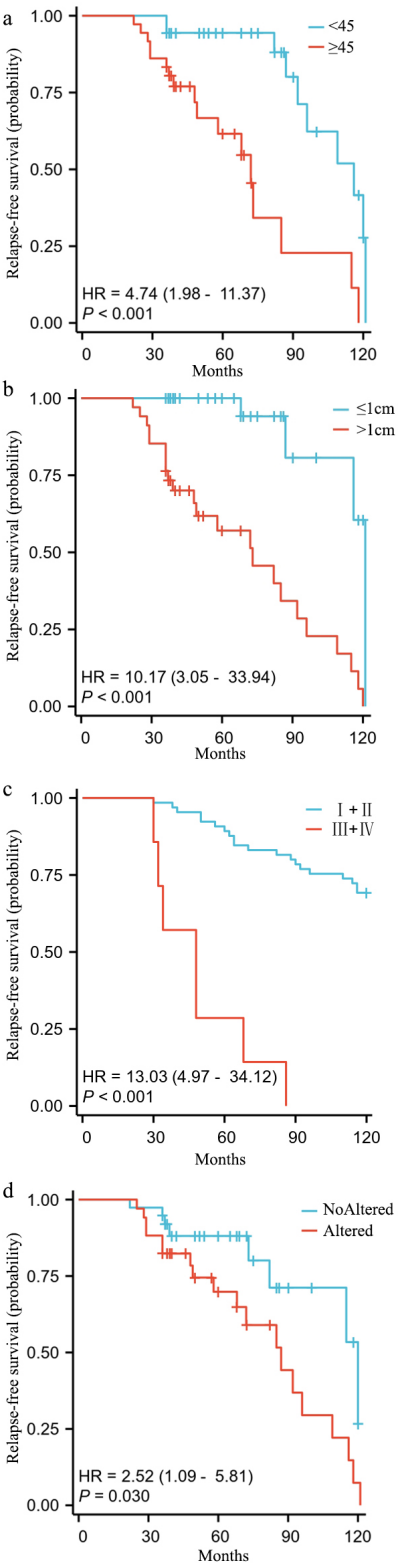
Kaplan-Meier survival analysis of relapse-free survival (RFS) in PTC. Kaplan-Meier survival curves illustrating relapse-free survival (RFS) in 72 PTC cases based on clinicopathological and genetic characteristics: **(a)** Age group (< 45 vs. ≥ 45 years); **(b)** Tumor size (≤ 1 cm vs. > 1 cm); **(c)** TNM stage (I+II vs. III+IV); **(d)** Presence of BRAF mutation (Altered vs. Non-Altered). Hazard ratios (HR) and p-values are shown for each comparison. Older age, larger tumor size, and advanced stage are significantly associated with worse RFS (p < 0.001). *BRAF* mutation shows a moderate but significant impact on prognosis (p = 0.030).

Given the observed mutation correlations, protein-protein interactions, and pathway alterations in thyroid cancer, we next investigated their clinical implications by assessing the association between key genetic alterations and survival outcomes. Kaplan-Meier survival analysis was performed to evaluate the impact of clinicopathological and molecular features on relapse-free survival (RFS) in the 72 PTC cases. Older age (≥ 45 years), larger tumor size (> 1 cm), and advanced TNM stage (III+IV) were significantly associated with reduced RFS (p < 0.001, **Figures 5a–c**). Additionally, the presence of *BRAF* mutations showed a moderate but significant correlation with poorer prognosis (HR = 2.52, p = 0.030, **Figure 5d**).

To further investigate the independent prognostic value of clinicopathological variables, Cox regression analysis was conducted (**Table 3**). Both univariate and multivariate analyses confirmed that age ≥ 45 years, advanced stage (III/IV), and tumor size > 1 cm were significant predictors of poor survival. Among these, age and TNM stage were identified as independent prognostic factors, reaffirming their critical role in thyroid cancer progression.

**Table 3:** Cox regression analysis of clinicopathological factors on survival in thyroid cancer.

Characteristics	Total(N)	Univariate analysis		Multivariate analysis	
		HR(95% CI)	P-value	HR(95% CI)	P-value
Age	72				
<45	33				
≥45	39	1.064 (1.030 - 1.100)	< 0.001	1.095 (1.054 - 1.137)	< 0.001
Gender	72				
Male	30				
Female	42	0.775 (0.349 - 1.721)	0.531		
Stage	72				
I	47				
II	19	2.059 (0.840 - 5.047)	0.114	6.060 (2.135 - 17.199)	< 0.001
III	2	7.874 (0.943 - 65.709)	0.057	36.002 (3.369 - 384.731)	0.003
IV	4	12.833 (3.715 - 44.325)	< 0.001	16.120 (4.123 - 63.022)	< 0.001
Smoke	72				
Yes	17				
No	55	1.446 (0.591 - 3.534)	0.419		
Alcohol	72				
Yes	62				
No	10	1.417 (0.560 - 3.585)	0.462		
Tumor location	72				
Left lobe	26				
Right lobe	35	0.883 (0.348 - 2.240)	0.794		
Bilateral	11	2.414 (0.863 - 6.754)	0.093		
Tumor Aspect ratio	72				
≤1	31				
>1	41	1.508 (0.641 - 3.547)	0.346		
Tumor nodules	72				
single	39				
multiple	33	0.945 (0.433 - 2.067)	0.888		
Tumor size (cm)	72				
≤1	38				
>1	34	10.167 (3.046 - 33.935)	< 0.001	10.167 (3.046 - 33.935)	< 0.001

HR, hazard ratio; Cl, confidence interval.

In addition to standard clinicopathological parameters, we evaluated whether thyroid hormone markers influenced survival outcomes (**Table 4**). No statistically significant associations were observed between pre- and post-operative thyroid function markers (TSH, PTH, FT3, FT4, TPOAb, TGAb) and survival (all p > 0.05). Post-operative FT4 showed no statistically significant association with prognosis (p = 0.07), though the observed trend may warrant further investigation in larger cohorts.

**Table 4:** Cox regression analysis of thyroid hormone on survival in thyroid cancer.

Characteristics	Total(N)	Univariate analysis		Multivariate analysis	
		HR(95% CI)	P-value	HR(95% CI)	P-value
Pre-operative TSH	72	0.868 (0.673 - 1.119)	0.27		
Pre-operative PTH	72	0.978 (0.951 - 1.006)	0.12		
Pre-operative FT3	72	1.082 (0.680 - 1.720)	0.74		
Pre-operative FT4	72	0.991 (0.930 - 1.055)	0.77		
TPOAb	72	0.999 (0.998 - 1.000)	0.22		
TGAb	72	0.999 (0.995 - 1.002)	0.46		
Post-operative CA	72	0.644 (0.044 - 9.407)	0.75		
Post-operative TSH	72	1.019 (0.984 - 1.055)	0.3		
Post-operative FT3	72	0.715 (0.307 - 1.669)	0.44		
Post-operative FT4	72	1.072 (0.994 - 1.155)	0.07	1.072 (0.994 - 1.155)	0.07

TSH, thyrotropin; PTH, parathyroid hormone; FT3, free triiodothyronine; FT4, free thyroxine; TPOAb, thyroid peroxidase antibody; TGAb, thyroglobulin antibody; HR, hazard ratio; Cl, confidence interval.

To assess the prognostic impact of recurrent genetic mutations, we performed Cox regression analysis on key driver mutations (**Table 5**). Interestingly, *BRAF* mutations were associated with significantly better survival (HR = 0.397, p = 0.03), which contrasts with the previously observed correlation with recurrence risk. This discrepancy suggests that while *BRAF* mutations may contribute to initial disease progression, they do not necessarily portend worse long-term survival. In contrast, *TERT*, *RET*, *TP53*, *CDKN2A*, and *NRAS* mutations did not show significant associations with survival outcomes (all p > 0.05), indicating that their impact may be more context-dependent or influenced by additional molecular factors.

**Table 5:** Cox regression analysis of mutations on survival in thyroid cancer.

Characteristics	Total(N)	Univariate analysis		Multivariate analysis	
		HR(95% CI)	P-value	HR(95% CI)	P-value
*BRAF*	72				
MUT	34				
WT	38	0.397 (0.172 - 0.915)	0.03	0.397 (0.172 - 0.915)	0.03
*TERT*	72				
MUT	24				
WT	48	1.756 (0.803 - 3.841)	0.16		
*RET*	72				
MUT	14				
WT	58	1.171 (0.337 - 4.067)	0.8		
*TP53*	72				
MUT	11				
WT	61	0.591 (0.250 - 1.398)	0.23		
*CDKN2A*	72				
MUT	8				
WT	64	1.620 (0.360 - 7.298)	0.53		
*NRAS*	72				
MUT	8				
WT	64	0.980 (0.291 - 3.292)	0.97		

MUT, Mutation; WT, wild-type; HR, hazard ratio; Cl, confidence interval.

To further assess the prognostic relevance of driver mutations in different thyroid cancer subtypes, survival analyses were conducted in the TCGA datasets. Disease-free survival (DFS) analysis in TCGA-PTC did not reveal any significant associations between *BRAF*, *NRAS*, *TERT*, *RET*, *PTEN*, or *BRCA2* mutations and DFS (**Supplementary Figure 3**). However, in TCGA-PDTC/ATC, mutations in *BRAF*, *TERT*, *TP53*, *CDKN2A*, and *BRCA2* were significantly associated with poorer overall survival (OS) (**Supplementary Figure 4**). In contrast, *RET*, *PTEN*, *ATM*, and *EP300* mutations did not demonstrate a significant impact on survival outcomes.

These findings highlight the differential prognostic roles of genetic alterations across thyroid cancer subtypes. While tumor size and TNM staging remain the strongest predictors of survival in PTC, *BRAF* mutations exhibit a complex role, contributing to recurrence risk but not necessarily leading to worse long-term outcomes. Meanwhile, in more aggressive forms of thyroid cancer, such as PDTC and ATC, *TP53*, *CDKN2A*, and *BRCA2* mutations are key contributors to poor prognosis, underscoring their role in tumor progression and therapy resistance. These observations reinforce the importance of integrating molecular profiling with clinical risk factors to refine prognostic stratification and guide personalized treatment strategies.

## 4 Discussion

This study of 72 papillary thyroid carcinoma (PTC) cases delineates a molecular landscape dominated by recurrent *BRAF* (47.2%), *TERT* (33.3%), and *TP53* (15.3%) mutations. *BRAF* and *TP53* mutations were strongly associated with recurrence (p < 0.001). Although *TERT* mutations approached but did not reach statistical significance (p = 0.050), their potential role was further investigation. Mutation interaction analysis revealed mutual exclusivity between *BRAF* and *RET*/*NRAS* (p < 0.01), reflecting distinct oncogenic pathways, and co-occurrence of *TP53* and *EP300*, suggesting chromatin remodeling defects in aggressive subtypes. Advanced TNM stage (HR = 13.03, p = 0.003) and tumor size (>1 cm) emerged as dominant prognostic factors, though *BRAF* paradoxically correlated with improved survival (HR = 0.397, p = 0.03). When contextualized against The Cancer Genome Atlas (TCGA) datasets, the elevated prevalence of *TP53* in poorly differentiated (PDTC) and anaplastic thyroid cancer (ATC) (28% vs. 0.8% in TCGA-PTC) underscores its role in tumor dedifferentiation (10). These findings collectively position *BRAF* as a key initiator of PTC, while *TERT* and *TP53* drive recurrence and progression, highlighting the associations between genetic alterations and clinical outcomes.

The high prevalence of *BRAF* V600E aligns with its established role in MAPK-driven tumorigenesis (30). However, its dual association—linked to recurrence risk but improved survival—raises critical questions. This discrepancy may reflect treatment biases, such as the potential for preferential use of targeted therapies in mutation-positive cases, which could confound survival outcomes. For instance, in radioiodine-refractory differentiated thyroid cancer (RAIR-DTC) patients treated with multi-kinase inhibitors (MKIs), those harboring the *BRAF* V600E mutation achieved better prognoses compared to patients with wild-type *BRAF* (31). Although we lack specific data on MKI use in our cohort, it is plausible that *BRAF* V600E-mutant patients were more likely to receive targeted therapies following recurrence, potentially contributing to improved survival by inhibiting MAPK signaling and other pathways involved in tumor progression. Another explanation could be the heterogeneity of *BRAF* mutations beyond the canonical *V600E* variant. While *BRAF* V600E is well-characterized and associated with aggressive tumor behavior and poorer outcomes, the clinical implications of non-V600E *BRAF* mutations (e.g., *BRAF* K601E and *BRAF* L597Q) remain poorly understood, with limited data on their prognostic and therapeutic relevance (32, 33). Furthermore, differences in post-recurrence management may also contribute to the observed survival benefit. *BRAF* V600E-mutant patients may have been more likely to receive aggressive treatment strategies following recurrence, such as repeat surgery, radiation therapy, or participation in clinical trials. However, due to the limitations of our retrospective study and the lack of comprehensive treatment data and subtype-specific information, we are unable to fully explain the paradoxical association between *BRAF* V600E mutation and survival. Future studies with larger cohorts and detailed treatment information are warranted to further explore this complex relationship.

In contrast, *TERT* mutations, despite their prevalence (33.3%), lacked a definitive association with recurrence (p = 0.050). This may be potentially due to cohort limitations, including the exclusion of PDTC/ATC subtypes where *TERT* is more prognostically impactful (34). Meanwhile, *TP53*’s intermediate prevalence (15.3%) bridges rates observed in differentiated (1%–3%) and anaplastic (80%) thyroid cancers (10), further supporting its established role as a key player in tumor progression and dedifferentiation. These observations underscore the need to interpret mutation profiles within both molecular and clinical contexts.

Mechanistically, the mutual exclusivity of *BRAF* with *RET/NRAS*, validated in TCGA-PTC, highlights divergent MAPK activation mechanisms. This biological dichotomy supports stratified therapeutic approaches: BRAF inhibitors for *BRAF*-mutant tumors versus RET inhibitors (e.g., selpercatinib) for *RET*-altered cases (35). Furthermore, the co-occurrence of *TP53* and *EP300* mutations suggests epigenetic dysregulation through chromatin remodeling defects (36), though functional studies are needed to confirm this hypothesis. Equally critical is the observed pathway evolution from MAPK dominance in PTC to PI3K/NOTCH activation in PDTC/ATC, which may explain the limited efficacy of BRAF inhibitors in advanced disease and underscores PI3K/mTOR inhibitors (e.g., everolimus) as promising therapeutic alternatives. These pathway-specific shifts emphasize the dynamic nature of thyroid cancer progression and the importance of tailoring therapies to molecular subtypes.

Clinically, TNM stage and tumor size remain the cornerstones of prognosis, but integrating *BRAF* and *TP53* status could refine risk stratification. For instance, *BRAF*/*TP53*-mutant stage I/II cases may necessitate intensified surveillance or adjuvant therapy. The survival advantage associated with *BRAF* mutations challenges conventional paradigms and warrants prospective validation to disentangle treatment-related confounders. Equally noteworthy is the lack of prognostic value in postoperative thyroid function markers (TSH, FT3, FT4), which emphasizes the primacy of molecular profiling over biochemical parameters in recurrence prediction. This finding aligns with some studies but contrasts with others that have reported ambiguous relationships between thyroid hormone levels and cancer recurrence (37). Therapeutic strategies must evolve to reflect this molecular heterogeneity: combining BRAF/MEK inhibitors (e.g., dabrafenib/trametinib) with radioiodine sensitizers may enhance efficacy in localized PTC, while advanced PDTC/ATC—characterized by PI3K/NOTCH activation—could benefit from pathway-specific agents such as everolimus or crenigacestat (38). Clinical studies have shown that the mTOR inhibitor everolimus exhibits antitumor activity in advanced differentiated thyroid cancer, although mTOR pathway mutations do not reliably predict response (39). Everolimus may also benefit PI3K/mTOR/Akt-mutated ATC, but genomic profiling is not yet a standard tool for guiding treatment decisions in thyroid cancer (40). The pan-PI3K inhibitor buparlisib failed to demonstrate significant efficacy in advanced FTC and PDTC; however, the observed decrease in tumor growth rate suggests potential benefits from combining PI3K and MAPK pathway inhibitors (41). Other PI3K/mTOR inhibitors, such as copanlisib and alpelisib, are also under investigation (42). A phase 1b study of the NOTCH inhibitor crenigacestat combined with the dual PI3K/mTOR inhibitor LY3023414 showed poor tolerability and limited clinical activity in advanced solid tumors (43). Despite these challenges, other NOTCH inhibitors continue to be explored in preclinical and clinical settings. Ultimately, personalized treatment strategies based on the molecular profiles of individual tumors will be crucial for optimizing therapeutic outcomes in thyroid cancer.

Despite these insights, several limitations warrant consideration. The retrospective design and modest cohort size (n = 72) limit statistical power, especially for detecting significant associations involving less frequent mutations such as *TP53* (15.3%) and *CDKN2A* (11.1%). These limitations may compromise the robustness of subgroup analyses and increase the risk of false negatives. Additionally, potential selection bias may exist, as evidenced by the slightly lower *BRAF* prevalence in our cohort compared to TCGA-PTC (47.2% vs. 50.0%).

Additionally, we acknowledge that treatment-related variables—such as adjuvant radioactive iodine (RAI) therapy, completeness of surgical resection, and other therapy types—can influence survival outcomes. However, due to the retrospective nature of our study and incomplete clinical documentation, these factors could not be included as covariates in our Cox regression analysis. Notably, all patients underwent radical thyroidectomy, which may help reduce variability in surgical management. Nonetheless, the absence of detailed treatment data limits our ability to fully account for potential confounding, and future prospective studies with comprehensive clinical annotation are warranted to clarify the impact of treatment modalities on prognosis.

Another limitation is the lack of subtype-specific data within our PTC cohort and the TCGA/MSK datasets. While PTC comprises various subtypes (diffuse sclerosing variant, tall cell variant, columnar cell variant, solid variant, and hobnail variant) with potentially distinct genetic profiles and clinical characteristics, our analysis was conducted on the overall PTC cohort given limited subtype-specific data. Future research should aim to incorporate subtype-specific analyses to provide a more nuanced understanding of the molecular mechanisms driving disease progression in different PTC subtypes.

Furthermore, the absence of functional validation precludes mechanistic validation of interactions such as *NRAS-PTEN-BRCA2*. Future research should prioritize multi-center prospective studies with standardized molecular profiling to validate *BRAF*’s dual prognostic role and clarify *TERT*’s subtype-dependent effects. Functional exploration of *TP53-EP300* and *NRAS-PTEN* interactions is critical to unravel their roles in chromatin remodeling and therapy resistance. Concurrently, clinical trials testing PI3K/NOTCH inhibitors in PDTC/ATC could bridge mechanistic insights to therapeutic application. Finally, while the use of TCGA and MSK datasets for cross-validation strengthens the generalizability of our findings, it is important to acknowledge that differences in patient populations, data collection protocols, clinical annotations, and sequencing platforms between these external datasets and our own cohort may introduce batch effects or reflect underlying clinical heterogeneity. These factors could influence observed mutation frequencies and their associations with clinical outcomes. Therefore, caution is warranted when interpreting cross-dataset comparisons, and future efforts should prioritize harmonized data integration across multi-institutional cohorts.

In conclusion, this study contributes to the understanding of thyroid cancer by reinforcing the clinical relevance of *BRAF*, *TERT*, and *TP53* in tumor initiation, recurrence, and progression, though further functional validation is needed. The mutual exclusivity of *BRAF* with *RET/NRAS* and its potential synergy with *TERT* underscore the complexity of mutation interactions in shaping tumor behavior. By integrating molecular profiling with conventional staging systems, clinicians may enhance the precision of risk stratification and tailor therapeutic strategies to individual molecular profiles. However, translational success hinges on validating these findings in larger cohorts and testing targeted therapies in rigorously designed trials. Such efforts will help advance precision oncology in thyroid cancer by validating mutation-informed strategies for risk stratification and therapeutic selection.

## Data Availability

The original contributions presented in the study are included in the article/**Supplementary Material**. Further inquiries can be directed to the corresponding author.
