# Supplementary material for: Mutation interactions of BRAF and TP53 define novel prognostic stratification and therapeutic implications in papillary thyroid carcinoma
Source: Front Endocrinol (Lausanne). 2025 Sep 29;16:1584618. doi: 10.3389/fendo.2025.1584618 (PMC12515616; doi:10.3389/fendo.2025.1584618)
Supplement: Supplementary file 1 [file DataSheet1.docx]

Supplementary Material

# Supplementary Figures and Tables

For more information on Supplementary Material and for details on the different file types accepted, please see [here](https://www.frontiersin.org/guidelines/author-guidelines#supplementary-material).

## Supplementary Figures


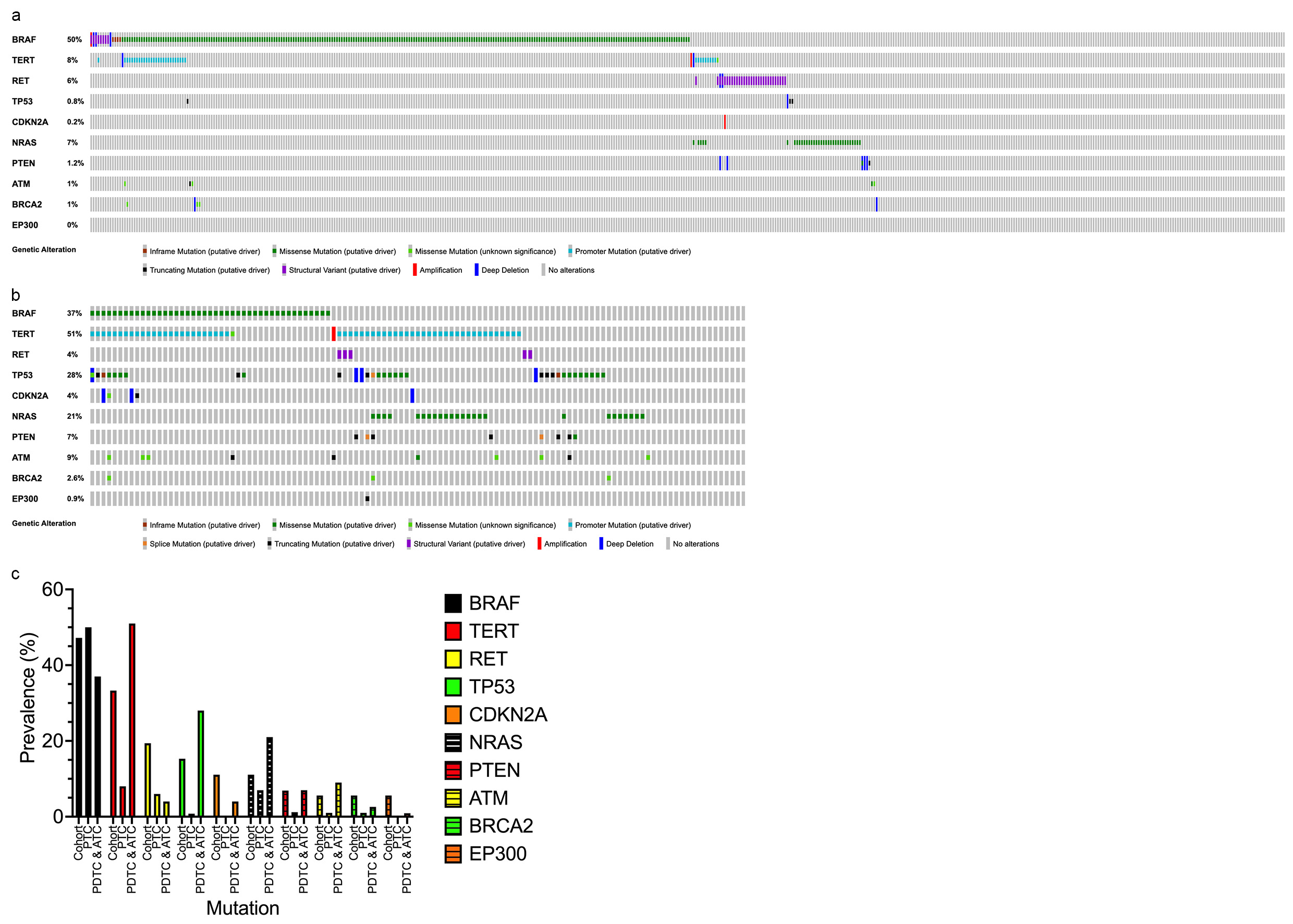


**Supplementary Figure 1.** **Mutation profiles in TCGA-PTC and TCGA-PDTC/ATC datasets.** Oncoprint diagrams showcasing the mutation distribution in TCGA datasets, including: (a) TCGA-PTC dataset; (b) TCGA-PDTC/ATC dataset; (c) Comparative mutation prevalence across the PTC cohort and TCGA datasets. Each panel displays the most frequently mutated genes, with different colors indicating mutation types. The bar plot in (c) quantifies the mutation frequency for key mutations in different thyroid cancer subtypes.

**
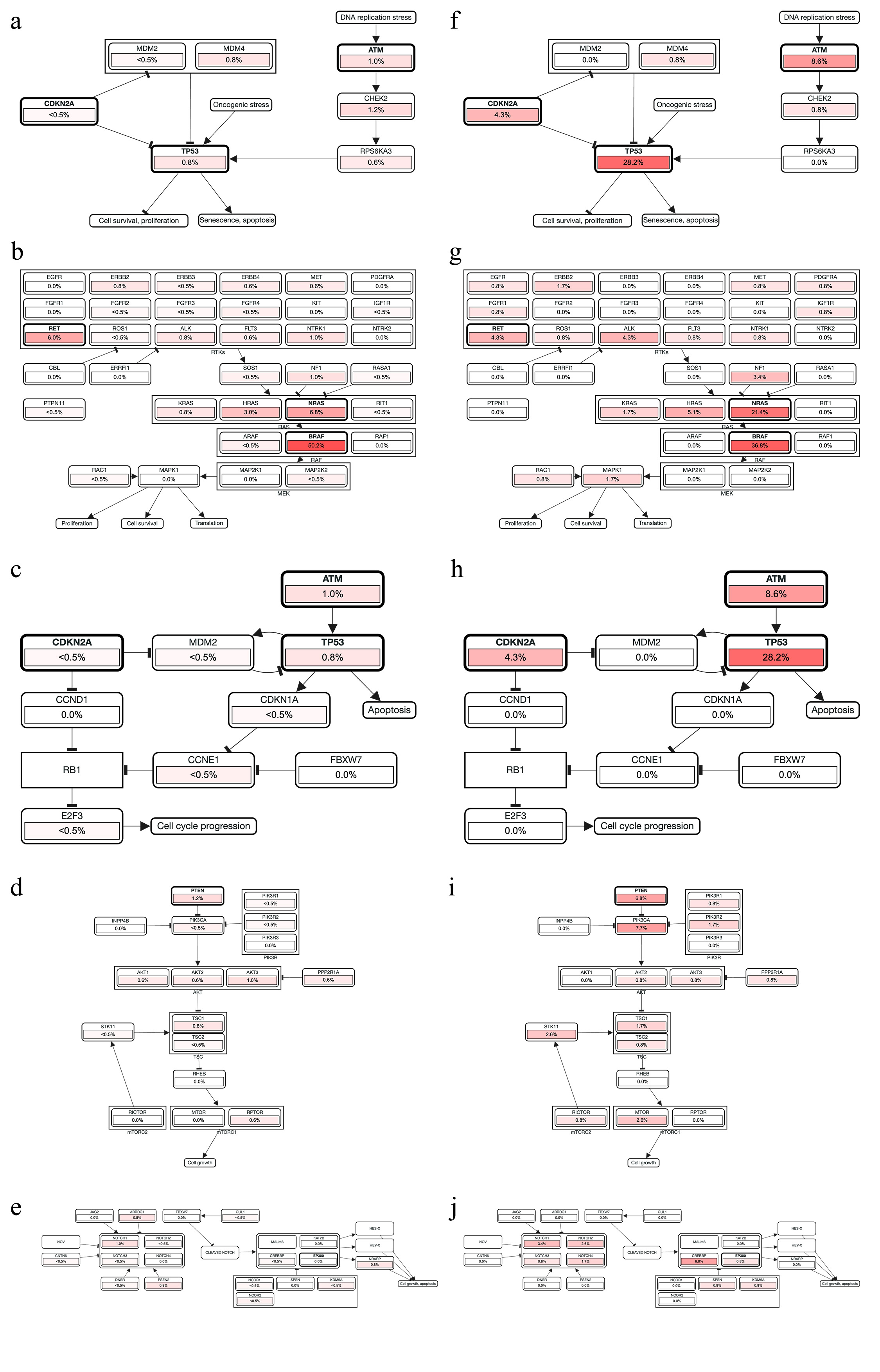
**

**Supplementary Figure 2. Comparative pathway analysis reveals distinct mutational impacts in TCGA-PTC and TCGA-PDTC/ATC datasets.** This figure illustrates key pathway alterations in PTC and PDTC/ATC, highlighting differences in tumor progression, therapeutic vulnerabilities, and prognosis. (a and f) Tumor suppressor regulation: PTC: Maintains intact *TP53/CDKN2A* function, contributing to controlled growth and better outcomes. PDTC/ATC: Displays frequent *TP53* and *CDKN2A* mutations, leading to genomic instability, aggressive progression, and resistance to therapy. (b and g) RTK-RAS signaling (MAPK pathway): PTC: Primarily *BRAF*-driven, supporting well-differentiated tumor characteristics. PDTC/ATC: Shifts toward *NRAS*-driven oncogenesis, which is associated with poorer differentiation and altered therapeutic response. (c and h) Cell cycle control: PTC: Exhibits minimal disruption of cell cycle regulators, indicating better proliferation control. PDTC/ATC: Shows deregulation of *CDKN2A* and *RB1*, resulting in uncontrolled cell cycle progression and reduced sensitivity to checkpoint inhibitors. (d and i) PI3K pathway activation: PTC: Rarely activated, with tumors primarily dependent on MAPK signaling. PDTC/ATC: Frequently altered, shifting tumors toward PI3K-driven survival mechanisms and therapy resistance. (e and j) NOTCH signaling: PTC: Largely intact, preserving normal differentiation programs. PDTC/ATC: Disrupted, favoring dedifferentiation, metastasis, and an aggressive phenotype.

**
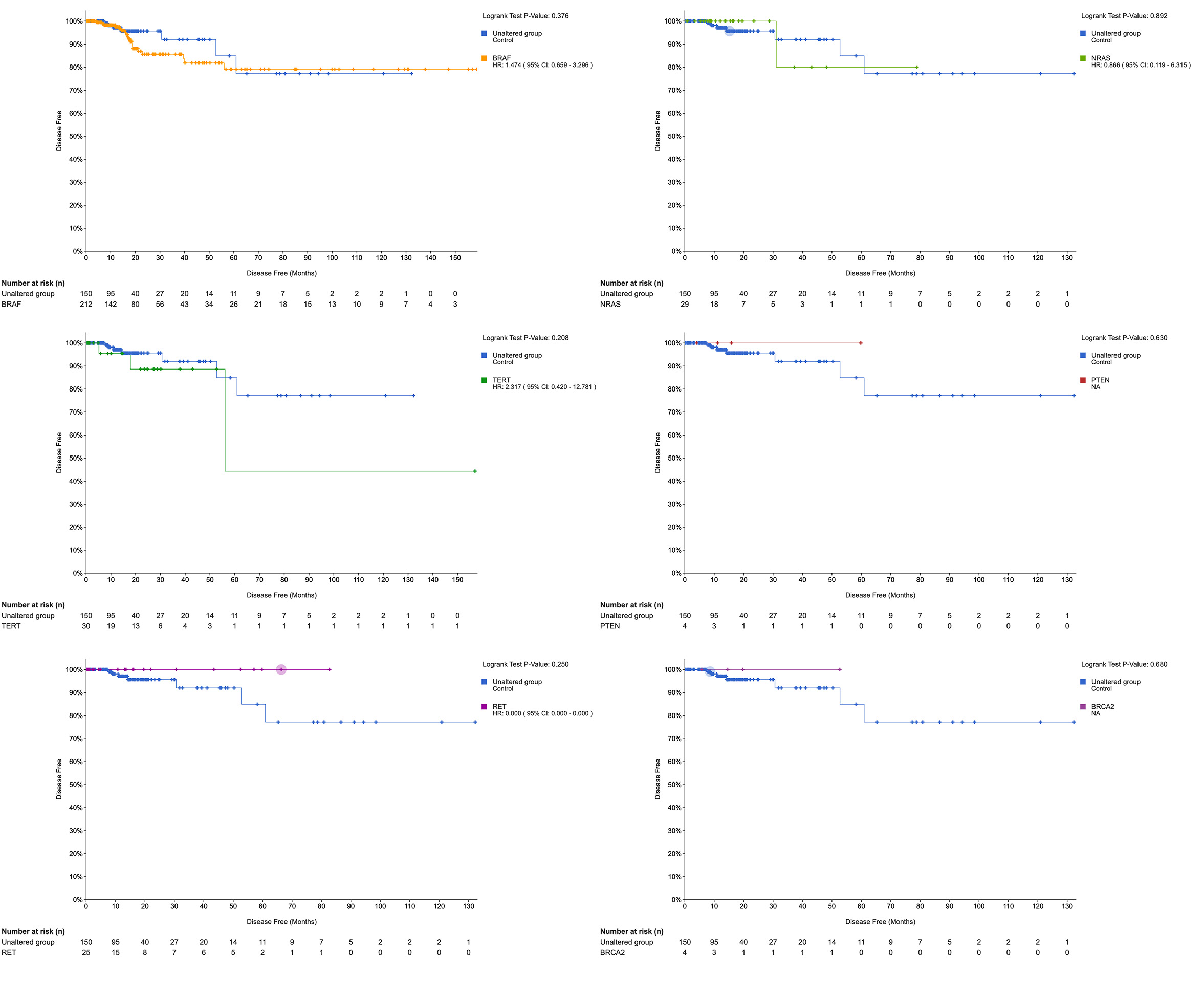
**

**Supplementary Figure 3.** **Kaplan-Meier analysis of disease-free survival (DFS) in TCGA-PTC.** Kaplan-Meier survival curves for disease-free survival (DFS) in TCGA-PTC cases, stratified by the presence of the indicated driver mutations. No significant association between these mutations and DFS was observed (all p > 0.05).

**
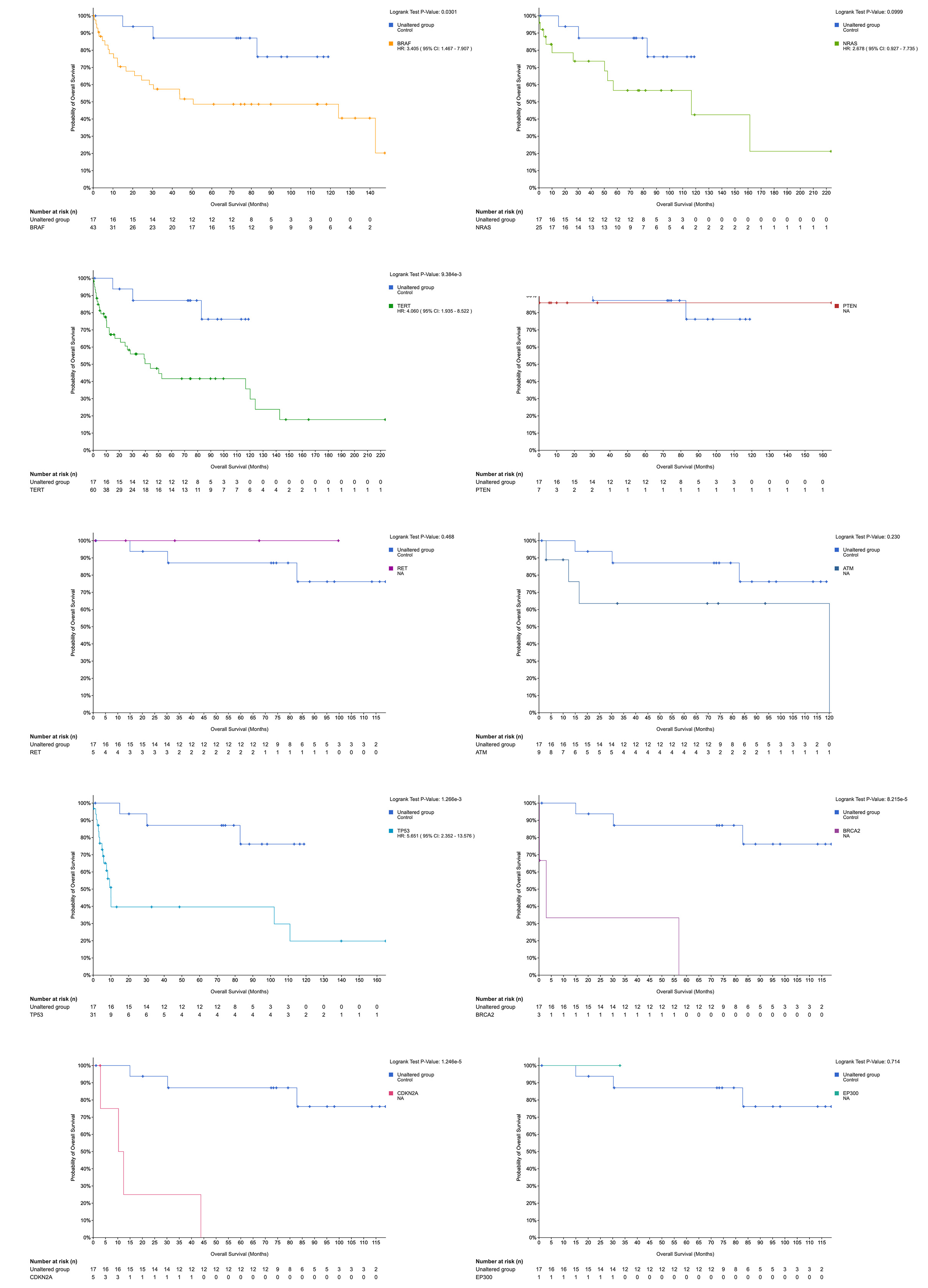
**

**Supplementary Figure 4. Kaplan-Meier analysis of overall survival (OS) in TCGA-PDTC/ATC.** Kaplan-Meier survival curves for overall survival (OS) in TCGA-PDTC/ATC cases, showing significant associations between *BRAF*, *TERT*, *TP53*, *CDKN2A*, and *BRCA2* mutations and worse OS (p < 0.05). No significant effect was observed for *RET*, *PTEN*, *ATM*, or *EP300* mutations.
